# Mitigation of Oxidized Oil Effects on Production Performances and Meat Quality of Broilers by Dietary Supplementation of Allicin

**DOI:** 10.3390/life14111432

**Published:** 2024-11-06

**Authors:** Arabela Elena Untea, Petru Alexandru Vlaicu, Iulia Varzaru, Alexandra Gabriela Oancea, Mihaela Saracila

**Affiliations:** Food and Feed Quality Department, National Research and Development Institute for Animal Biology and Nutrition, 077015 Balotesti, IF, Romania; iulia.maros@ibna.ro (I.V.); alexandra.oancea@ibna.ro (A.G.O.); mihaela.saracila@ibna.ro (M.S.)

**Keywords:** broilers, meat quality, oxidized oil, garlic, antioxidants, lipids, allicin

## Abstract

The objective of this study was to evaluate the effects of dietary oxidized oil and allicin (two different dietary sources) as natural antioxidants on the growth performance and meat quality of broilers. A total of 200 one-day-old Ross 308 broilers were randomly divided into four dietary groups (50 birds/group). The experimental groups (OO—oxidized oil; OOA—oxidized oil and allicin; OOG—oxidized oil and garlic leaves) differed from the control one by the presence of oxidized oil in their dietary structure (peroxide value 9.07 (OO, OOA and OOG groups) vs. 1.70 (C group) meq active oxygen/kg). The diets given to the experimental groups differed from each other by the presence of allicin (100 mg/kg inclusion rate as extract (OOA) and 0.5% as garlic leaf powder (OOG)). At the end of the experiment, six animals/group were slaughtered, meat samples (breast and thigh) were collected, and nutritional value was established. The results showed that the allicin included in the experimental diet did not influence the proximate composition of breast meat (crude protein, fat, ash, and dry matter). The fatty acid profile was determined for each group of samples; a significant decrease in omega 3 FAs was noticed between the C group and the E groups (3.27% vs. 1.46%, 1.60%, and 1.56%) in breast meat samples, and a corresponding increase was noticed in saturated fatty acid (SFA) concentrations. Health indices with implications for atheroma and thrombus formation and cholesterol level were negatively affected by the presence of oxidized oil in the experimental diets, but the allicin extract supplement appeared to mitigate its influence. A positive influence of the dietary supplement was noticed on antioxidant capacity and polyphenol concentrations determined in breast and thigh samples under allicin supplement influence. The results of the current study revealed that the use of low oxidized oil in broilers diets did not affect productive performance. The nutritional quality of meat (breast and thigh) was negatively influenced by the presence of oxidized oil, but allicin supplements (extract or garlic leaves) improved lipid quality indices and antioxidant potential.

## 1. Introduction

Reuse, reduce, recycle, i.e., the 3Rs from the circular economy concept, in the agri-food industry are closely related to the reincorporation and valuation of waste and by-products. Recycling, viewed as recovery and reprocessing, is one of the main elements of the circular economy [1]. Before recycling, other components must be considered such as redesigning products using more waste materials and fewer raw (virgin) materials, making progress in reducing the concept of waste. Within the strategy of transforming waste materials into valuable products, used vegetable oil occupies one of the leading roles. The main component of this family is waste cooking oil, as it is known that frying is the main cooking method in many countries around the world, and the European Union production is estimated to be 1 million tons/year [2]. The reconversion of the waste implies three main industrial directions: fuel production, additives for asphalt, or animal feed. Lipids are the richest energy source in animals’ diets, providing over two times more energy compared with carbohydrates or proteins. Frying food in heated oil exposes the unsaturated fatty acids to thermal oxidation processes, resulting in free radicals, aldehydes, ketones, and other degradation products, which are chemical compounds considered harmful for human and animal health [3]. The reactions of oxidation products with fat soluble vitamins and also with proteins from animals’ diets negatively affect the nutritional value of feeds. By reviewing the studies on dietary oxidized oils used in poultry nutrition, some authors [4] concluded that the rancid odors of used oils affect palatability and determine poor growth performances and nutrient absorption. Oxidative stress effects are evidenced, and fat-soluble vitamins are affected [5]. Antioxidants are powerful chemical compounds in counteracting the effects of lipid oxidation in several ways like scavenging free radicals, decomposing peroxides, or inhibiting the oxidation catalysts (metals). The selection of antioxidants for preventing the oxidative damage must consider the antioxidant properties and the action mode [6].

Garlic is a well-known herb in traditional medicine, but also its benefic role in poultry production is recognized. According to the study of Aarti et al. [7], it contains 33 sulfur compounds, enzymes, amino acids, and minerals. Allicin is the most abundant thiosulfonate in garlic structure, presenting many beneficial attributes like antimicrobic, anti-inflammatory, and antitumor properties. The beneficial effects of allicin include a reducing potential of triglycerides and cholesterol in blood and liver, adjuvant in cardiovascular disease, bacterial development inhibition, and a positive effect on oxidative stress parameters. Improving broiler performances such as growth and feed conversion rate are the beneficial effects of garlic in poultry diets mentioned in the scientific literature [8].

In this context, the aim of this study was to evaluate the effects of different dietary allicin (extract or garlic leaf) supplements on the production performances and nutritional quality of the meat of broilers fed oxidized oil.

## 2. Materials and Methods

### 2.1. Experimental Design and Growth Performance Monitoring

A nutrition experiment took place under regulations provided by legislation regarding animal welfare (Romanian documents 206/2004, 43/11.04.2014 and Directive 2010/63/EU) The experimental procedures were approved by the Ethical Commission of the National Research and Development Institute for Biology and Animal Nutrition, protocol No. 4992/11.10.2023, of the PN 23.20-03.01/2023 project. Experimental conditions were designed according to the breeding guide of the hybrid and sanitary veterinary regulations.

A total of 200 one-day-old Ross 308 broiler chickens were randomly divided into 4 dietary groups (50 animals/group). The experimental groups differed from the control one by the presence of oxidized oil in their structure (peroxide value 9.07 (OO—oxidized oil (positive control group); OOA—oxidized oil and allicin; OOG—oxidized oil and garlic leaves) vs. 1.70 (fresh oil group—FO (negative control group)) meq active oxygen/kg). The experimental groups differed from each other by the presence of allicin in the OOA group (100 mg/kg inclusion rate as extract (Swanson, USA, 1% allicin extracted from garlic bulb) and 0.5% as garlic leaf powder in the OOG group (local producer). The experimental hall where the broilers were housed simulated the semi-intensive system condition. The broilers were raised on the floor using a permanent wood shaving litter with a thickness of 10–12 cm. Each experimental group was housed in a growth chamber, designed to provide adequate space for development until 42 days of age, with a stocking density of 16 broilers/m^2^. Environmental conditions, including temperature, humidity, and ventilation, were monitored daily using a ViperTouch control system. The initial temperature was set at 32 °C on day one and was reduced by 2 °C per week thereafter. The lighting regimen consisted of 23 h of light followed by 1 h of darkness and gradually decreased until it reached 16L:8D. Feed and water were supplied ad libitum throughout the experiment. At the end of experimental period (14–42 days), from each group, 6 animals were randomly selected and slaughtered by cervical dislocation. Breast and thigh meat were collected and stored at −80 °C for further analysis.

At the beginning and at the end of experiment, every animal was weighed and recorded to establish the initial and final body weight (BW, g). Average daily feed intake (ADFI, g feed/broiler/day) was considered to be the difference between the offered and rejected feed and it was recorded daily. For the average daily weight gain (ADWG, g) productive parameter, broiler weight was divided by the number of experimental days, and the ratio between feed consumed and total weight represents the feed conversion ratio (feed/gain (g:g)). In Table 1 are presented details regarding feed structures used in the experiment.

The production efficiency parameters such as the European Production Efficiency Factor (EPEF) and the European Broiler Index (EBI) were calculated according to Vlaicu et al. [9].

### 2.2. Analytical Characterization of Plants, Feeds, and Animal Origin Samples

#### 2.2.1. Proximate Composition and Trace Mineral Content

For the crude protein determination, the Kjeldahl reference method was employed, using a semiautomatic Kjeltek auto 1030-Tecator (FOSS Tecator AB, Höganäs, Sweden). Continuous extraction in solvent, followed by fat measurement with Soxhlet after solvent removal (FOSS Tecator AB, Höganäs, Sweden), was used for crude fat determination. Dry matter and crude ash were determined by gravimetric methods using a BMT model ECOCELL Blueline Comfort (Neuremberg, Germany) and Nabertherm Labotherm L15/11/P320 Comfort (Bremen, Germany). The methods are described elsewhere [10].

Flame atomic absorption spectrometry (FAAS) for determination and microwave digestion for sample preparation were used for trace mineral determination, as described by Untea et al. [11]. The equipment used was Thermo Electron-SOLAAR M6 Dual Zeeman Comfort (Cambridge, UK).

#### 2.2.2. Fatty Acid Profile Determination

A Perkin Elmer Clarus 500 (MA, Massachusetts, United States of America, USA) gas chromatograph was used for fatty acid quantification in vegetal materials and animal origin samples. The method consists of the transformation of fatty acids into methyl esters, their separation, and their identification through a chromatographic method and reference chromatograms provided by standards. The gas chromatograph possesses a flame ionization detector (FID) and a capillary separation column with a high stationary polar phase TRACE TR-Fame (Thermo Electron, MA, USA), measuring 60 m × 0.25 mm × 0.25 μm film. The method was described by Turcu et al. [12].

Atherogenicity index (AI), thrombogenicity index (TI), hypercholesterolemic fatty acids (OFAs), hypocholesterolemic fatty acids (DFAs), peroxidisability index (PI), iodine value (IV), calculated oxidizability (COX), and oxidative susceptibility (OS) lipid indices were calculated according to formulas described by Varzaru et al. [13].

#### 2.2.3. Total Polyphenol Content and Antioxidant Capacity Evaluation

For the total polyphenol content, the sample solution reacted with Folin–Ciocalteu reagent in the presence of sodium carbonate, and the absorbance was recorded at 732 nm and converted into mg Gallic acid equivalents per gram sample. Antioxidant capacity was evaluated using the DPPH method. Both analytical methods are described by Untea et al. [14], and the determination equipment was a spectrophotometer (Jasco V-530, Servo Co., Ltd., Tokyo, Japan).

### 2.3. Statistical Analysis

For performance parameters (BWi, BWf, and ADWG), every broiler was considered an experimental unit while ADFI and the feed conversion ratio were calculated, considering the box as an experimental unit for statistical analyses. For statistical interpretation, every broiler was considered an experimental unit. The experimental data were statistically analyzed using XLSTAT software, desktop version (2023.5) produced by Addinsoft (New York, NY, USA), where *p* values below 5% were considered significant. All analytical determinations were performed in triplicate.

## 3. Results

The analytical composition and antioxidant capacity of allicin extract (AE) and garlic leaves (GL) are presented in Table 2. The allicin extract was presented as pills which were crushed and transformed into powder, and the dry matter was determined to have an important value close to 100%. The garlic leaves were purchased as fresh material and the dry matter determined was almost eight times smaller than AE. The crude protein presented similar values, but the fiber and ash content were higher in the case of leaves. Iron and manganese concentrations in leaves presented important values compared to allicin, and the same observation was valid for antioxidants. Stearic acid was a component of the extract (manufacturer declaration) and, in consequence, the saturated fatty acids were the main class of lipids present in the AE structure. Garlic leaves possessed a different profile, in which PUFAs were the main components and important concentrations of omega 3 FAs were determined.

Growth performance parameters are presented in Table 3. No statistical differences were noticed between groups for body weight (initial and final) or ADFI or ADWG. An exception was registered, in the case of the feed/gain ratio, with a significantly decreased value being calculated for the group supplemented with allicin extract. The production efficiency factors (European Production Efficiency Factor (EPEF) and European Broiler Index (EBI)) registered the lowest values for the OO group, proving the effect of oxidized oil in diets, and the highest values for the OOA group, with the presence of allicin elevating the negative effects of oil.

Samples of breast and thigh meat were analyzed, their chemical composition was established, and the results are presented in Table 4. The proximate composition of breast samples was not influenced by the dietary supplements administrated. Significantly increased concentrations of iron were determined in the samples belonging to the oxidized oil groups (OO groups). Increased fat concentrations were detected in thigh samples accompanied by low levels of protein in groups supplemented with allicin (extract or garlic leaves), but the differences between protein concentrations were only numerical.

The fatty acid profiles of the considered tissue samples are presented in Table 5 and Table 6. Increased concentrations of saturated fatty acids and significantly decreased omega 3 concentrations were registered for breast tissue samples.

No significant differences were noticed in lipid classes of thigh meat (Table 6). A significant decrease in some saturated fatty acids was calculated (caprylic, capric, or myristic acids), but the main contributors like palmitic or stearic acids did not differ between groups. Long-chain omega fatty acids reacted on dietary supplements, with docosatrienoic, eicosapentaenoic, and docosahexaenoic acids being positively influenced by allicin (extract or garlic leaves).

The lipid quality indices calculated for both anatomical parts considered are presented in Table 7. A significantly decreased ratio was calculated for the OO group compared with the control when PUFAs were related to SFAs. The ratio Ω6/Ω3 was almost twice lower for the group with fresh oil compared with the experimental groups fed with oxidized oil, but a significantly increased conversion rate of omega 3 FAs into similar long-chain FAs was recorded for the same groups. The atherogenicity index and hypercholesterolemic and hypocholesterolemic fatty acids were influenced by the allicin extract, presenting similar values to the control group. Oxidative susceptibility was increased for the control group compared to others, due to the high amount of omega 3 FAs detected in breast samples for this group.

**Figure 1:**
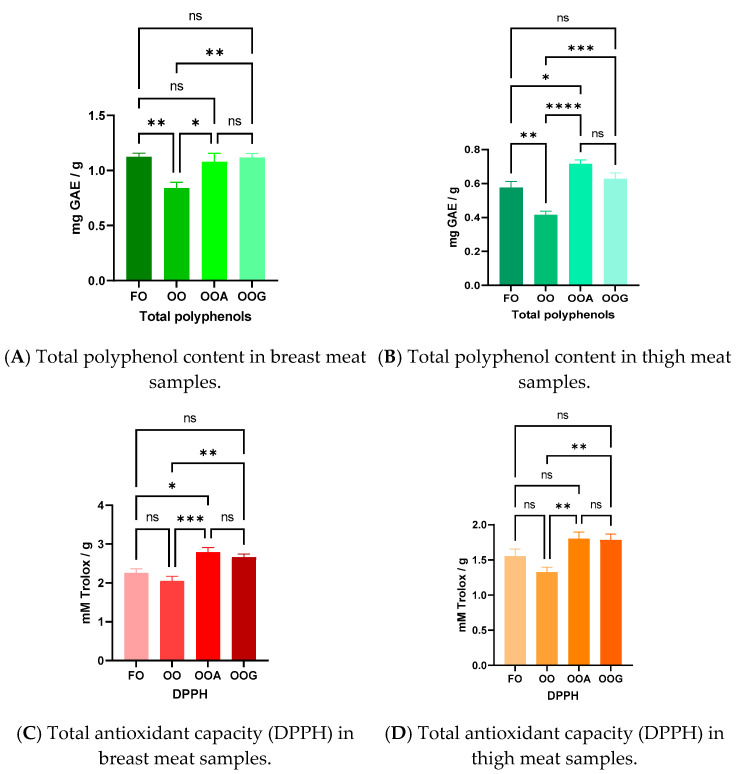
Total polyphenol content (**A**,**B**) and antioxidant capacity (**C**,**D**) of breast and thigh meat. FO—group fed with fresh oil diet; OO—group fed with oxidized oil; OOA—group fed with oxidized oil and allicin extract; OOG—group fed with oxidized oil and garlic leaves. * *p* < 0.05, ** *p* < 0.001, *** *p* <0.0001, **** *p* < 0.00001, significance level; ns—non significant.

Figure 1 revealed a significantly decreased total polyphenol content for the group which received oxidized oil without allicin supplement (extract or garlic leaves). In thigh samples, the dietary supplements led to increased polyphenol deposition, with the allicin extract-supplemented group registering increased concentrations compared with the control. Antioxidant capacity followed the same pattern, with the dietary supplements producing a positive effect on the oxidative status of breast and thigh meat.

## 4. Discussion

### 4.1. Chemical Composition of Garlic

Allicin is a sulfur component which is produced by the enzymes allin and alliinase when garlic is crushed [15]. Other non-sulfur compounds present in the garlic chemical structure provide a synergistic biological effect on human and animal bodies, exerting health benefits [16].

In a study conducted on garlic leaves, the authors reported results for different Polish varieties of garlic as follows: dry matter 10–15%, crude fat 1.6–5.6%, crude ash 8.9–14.1%, and crude protein 13.7–35.7%. The mineral content declared was 34–230 mg/kg iron and 9–58 mg/kg zinc. Our results regarding the chemical composition of garlic leaves are in the same range of values as the wild variety reported by the cited authors [17]. Other authors studied black garlic (fresh, oven dried, and encapsulated) obtained by various methods, and the total polyphenols were between 10.30 and 14.40 GAE mg/g, a range within which the values reported in the present study also fall [18].

As in our findings, palmitic and linoleic acids are the main contributors of garlic lipid structure, which determine the total SFA and omega 6 content [19]. The profile determined in the current study proved that garlic leaves are a valuable source of omega 3 fatty acids, especially linolenic acid. Due to its high content of omega 3 fatty acids, the ratio Ω6/Ω3 presented a minimum value, with maximum importance for health status.

### 4.2. Growth Performance

Vegetable oils are used in the poultry nutrition as energy source, but they are prone to oxidation; however, the type of oil and the degree of oxidation lead to different impacts on animal performances. A similar study was conducted using oxidized oil at a moderate level (PV = 7.5 meq/kg) and the productive parameters were depressed [20]. No negative impact on performances was noticed in our study (PV = 9.07 meq/kg), similar to others who reported no significant differences between groups even if they used different degrees of oxidation of oils (PV = 25 or 56 or 73 meq) [21], or when fresh oil was partially (0–100%) replaced with oxidized oil (PV = 68.4 meq/kg) [22].

The effects of allicin as extract or garlic supplementation in broiler nutrition were reported previously by other researchers. For example, Fan et al. [23] reported a similar conclusion to ours, namely, that the feed conversion ratio decreased significantly under allicin extract influence, using 400 mg/kg allicin in their experimental diet. In another study where broilers’ diets were supplemented with allicin extract, a dose of 150 mg/kg led to an increased body weight gain and had no influence on the feed-to-gain ratio [24]. The authors showed that garlic extract modulates glucose homeostasis and exerts antidiabetic properties, preventing obesity and metabolic disorders. Some of these properties can be related to skeletal muscle development and, overall, to animals’ productive parameters. A positive influence on the feed conversion ratio was also obtained by introducing garlic powder (3%) in the diets of broilers. The use of fresh garlic in diets contributes to improving feed efficiency and body weight [25].

### 4.3. Proximate and Mineral Composition

Studies regarding the effects of oxidized oils on broilers revealed that protein and fat digestibility are not affected compared with birds fed fresh oil diets [4]. Our results showed no significant differences between groups for chemical composition determined in breast meat and a higher content of fat in thigh meat, but under the influence of allicin supplements. In a nutrition study on broilers, where the effect of garlic powder extracted from bulbs and incorporated into diets at 1, 3, and 5% inclusion level was tested, moisture and crude ash contents in thigh meat were not affected by the treatments. Crude protein level was increased, and crude fat was decreased in the garlic-supplemented groups. The authors presumed that the effects could be attributed to the decreased accumulation of fat in the liver, with the biosynthesis of lipids being affected by the supplements [26].

A study on the relationship between the oxidation degrees of diets, the oxidative stress induced to the animals and biochemical reactions in muscle showed significant effects on lipid and protein oxidation. Meat contains pro-oxidative heme proteins like myoglobin in which iron is an initiation factor, and the authors proved that non-heme iron increased in meat, as it was released due to myoglobin denaturation. The results presented in the current study showed increased levels of iron both in breast and thigh meat, as a possible consequence of the oxidative stress produced by the oxidized oil present in the experimental diets [27]. Only the supplementation of diets with allicin led to results similar to that of animals which consumed fresh oil.

### 4.4. Fatty Acid Profile

Saturated fatty acids presented significantly lower concentrations in breast meat samples for the control group, compared with the groups which received oxidized oil, with palmitic acid being the main contributor to this class of acids. Oleic acid was influenced by the degraded oil from the diets and presented decreased concentrations in the oxidized oil groups compared to the control. As a consequence, the total MUFAs were significantly decreased in the experimental groups. Degraded oil from the diets negatively influenced the omega 3 concentrations in breast meat. But omega 6 concentrations, particularly arachidonic acid, registered increased concentrations under allicin extract influence (OOA group). As a result, PUFA concentrations were increased in the mentioned group compared to the groups which did not receive any form of allicin (FO and OO). Due to the increased omega 3 concentrations, Ω6/Ω3 presented the most important value in the fresh oil group (FO). Other studies noticed that the fatty acid profile of meat is influenced by the oxidized oil present in the diet, with increased concentrations of SFAs and decreased concentrations of PUFAs being reported [28,29]. Increased concentrations of omega 6 fatty acids were obtained for the groups supplemented with allicin (OOA and OOG), due to the important levels of linoleic acid. A possible explanation could be that allicin (extract or garlic leaves) stimulates scavenging enzyme activity and reduces unsaturated fatty acid oxidation [30].

### 4.5. Lipid Quality Indices

The nutritional indices determined in breast samples were related to the increased linolenic acid concentrations in the control samples compared to groups which received oxidized oil in their diets. In fact, the ratio of Ω6 to Ω3 registered a minimum value for the FO group, but an increased linolenic acid level did not lead to a maximum conversion rate to long-chain fatty acids, and the ratio between them recorded a significantly decreased value compared to other groups. An increased conversion rate for long-chain omega 3 fatty acids and a decreased conversion rate for similar omega 6 acids were recorded for the group supplemented with garlic leaves. Long-chain PUFAs (EPA and DHA) are considered essential fatty acids for human health with beneficial implications for cardiovascular and other diseases [31]. It has been demonstrated that long-chain PUFAs are biosynthesized with a low yield in mammals and birds [32], with omega 3 FAs from diets (with linolenic acid as the main component) being proportionally found in animal tissues. On the other hand, oxidative stress depresses lipid liver synthesis [33], an effect also observed in our study (omega 3 FA concentrations decreased under oxidized oil influence compared with the group fed with fresh oil). Untea et al. [34] observed that a powerful source of antioxidants can lead to the increased bioconversion of long-chain PUFAs from their precursors. In our study, compared with the OO group, higher values were calculated in thigh samples for the allicin-supplemented groups, but garlic (which may be a combined source of antioxidants) produced a significant effect. Health indices with implications for atheroma and thrombus formation (AI and TI) and cholesterol level [12,35] were evaluated in the current study. They were negatively affected by the presence of oxidized oil in the experimental diets, but the allicin extract supplement appeared to mitigate its influence, with no significant differences being recorded compared to the control in terms of AI, OFAs, and DFAs. The oxidative indices were increased in the control group compared to others; this observation is related to the increased omega 3 concentrations determined for this group in breast samples.

### 4.6. Antioxidants

Nutritional studies on farm animals have demonstrated that bioactive compounds in diets can be efficiently incorporated into their tissues. This nutritional strategy could ensure the intake of biologically active compounds with low availability from plants, herbs, or other plant materials through animal foods enriched in these compounds [36]. As expected, the oxidative stress induced by the presence of oxidized oil in the experimental diets affected the antioxidant capacity and total polyphenol concentrations of breast and thigh samples. Statistically, the antioxidant capacity and total polyphenol concentrations determined in the groups fed with oxidized oil and supplemented with garlic leaves were similar to those of the group fed with fresh oil. The study of the antioxidant performance of chickens fed with allicin-supplemented diets showed a positive effect on antioxidant capacity and specific enzymes determined in blood samples [37]. An increase in antioxidant status was also observed by [38] when garlic extract (10 and 15 g/kg) was experimentally added to chicken diets.

## 5. Conclusions

The results of the current study revealed that the use of low oxidized oil (PV 9.7 meq/kg) in broilers’ diets did not affect productive performances. The nutritional quality of meat (breast and thigh) was negatively influenced by the presence of oxidized oil, but allicin supplements (extract or garlic leaves) improved lipid quality indices and antioxidant potential.

## Figures and Tables

**Table 1:** Dietary compound feeds for broiler chickens in the grower and finisher phases.

Ingredients, % (As Feed Basis)	Starter Phase	FO	OO	OOA	OOG	FO	OO	OOA	OOG
		Grower Phase (14–28 Days)				Finisher Phase (29–42 Days)			
Corn	44.50	38.45	38.45	38.44	38.45	47.95	47.95	47.94	47.95
Soybean meal	35.11	27.81	27.81	27.81	27.81	13.26	13.26	13.26	12.76
Wheat	8.86	19.00	19.00	19.00	18.50	20.00	20.00	20.00	20.00
Corn gluten	3.00	4.00	4.00	4.00	4.00	8.00	8.00	8.00	8.00
Vegetal fresh oil	3.42	5.50	-	-	-	5.50	-	-	-
Vegetal oxidized oil	-	-	5.50	5.50	5.50	-	5.50	5.50	5.50
Garlic leaves	-	-	-	-	0.50	-	-	-	0.50
Allicin	-	-	-	0.01	-	-	-	0.01	-
L—Lysine—HCl	0.26	0.32	0.32	0.32	0.32	0.49	0.49	0.49	0.49
DL-Methionine	0.30	0.18	0.18	0.18	0.18	0.20	0.20	0.20	0.20
L-Threonine	0.10	0.20	0.20	0.20	0.20	0.20	0.20	0.20	0.20
Calcium carbonate	1.37	1.65	1.65	1.65	1.65	1.60	1.60	1.60	1.60
Monocalcium phosphate	1.51	1.54	1.54	1.54	1.54	1.45	1.45	1.45	1.45
Salt	0.30	0.30	0.30	0.30	0.30	0.30	0.30	0.30	0.30
Choline	0.37	0.05	0.05	0.05	0.05	0.05	0.05	0.05	0.05
Premix for broilers	1.00	1.00	1.00	1.00	1.00	1.00	1.00	1.00	1.00
Total ingredients	100	100	100	100	100	100	100	100	100
Calculated nutritional composition									
Metabolizable energy, kcal	2975	3100				3150			
Dry matter, %	87.12	86.02				86.26			
Crude protein, %	21.41	21.00				19.00			
Crude fiber, %	3.88	3.37				2.61			
Crude fat, %	5.34	7.91				8.29			
Ash, %	3.12	4.77				3.99			

Premix composition per kg feed: 11.000 IU/kg vitamin A; 2.000 IU/kg vitamin D3; 27 IU/kg vitamin E; 3 mg/kg vitamin K; 2 mg/kg vitamin B1; 4 mg/kg vitamin B2; 14.85 mg/kg pantothenic acid; 27 mg/kg nicotinic acid; 3 mg/kg vitamin B6; 0.04 mg/kg vitamin B7; 1 mg/kg vitamin B9; 0.018 mg/kg vitamin B12; 20 mg/kg vitamin C; 80 mg/kg Mn; 80 mg/kg Fe; 5 mg/kg Cu; 0.60 mg/kg Zn; 0.37 mg/kg Co; 1.52 mg/kg I; 0.18 mg/kg Se. Allicin supplement was added to the premix at the inclusion level of 100 mg/kg. FO—fresh oil group; OO—oxidized oil; OOA—oxidized oil and allicin; OOG—oxidized oil and garlic leaves.

**Table 2:** Nutrient composition of allicin supplements (extract and garlic leaves).

ITEM	AE	GL	SEM	*p* Value
Proximate composition				
Dry matter (%)	90.61 ^a^	11.25 ^b^	0.842	0.021
Crude protein (%)	14.06 ^b^	14.61 ^a^	0.115	0.021
Crude fat (%)	6.76 ^a^	1.79 ^b^	0.309	0.001
Crude fiber (%)	6.66 ^b^	18.87 ^a^	0.476	0.001
Crude ash (%)	3.50 ^b^	10.22 ^a^	0.456	0.001
Trace mineral composition				
Copper (mg/kg)	2.09	2.22	0.115	0.288
Iron (mg/kg)	62.26 ^b^	261.93 ^a^	12.35	0.001
Manganese (mg/kg)	3.50 ^b^	30.63 ^a^	1.242	0.001
Zinc (mg/kg)	13.49 ^b^	17.09 ^a^	0.654	0.015
Antioxidants				
TP (mg/g GAE)	0.17 ^b^	11.32 ^a^	0.127	0.001
AC (mM trolox)	6.92 ^b^	54.73 ^a^	0.829	0.001
Fatty acid composition				
SFAs (%)	92.07 ^a^	38.31 ^b^	0.016	0.001
MUFAs (%)	2.96 ^b^	8.32 ^a^	0.070	0.001
PUFAs (%)	4.83 ^b^	53.38 ^a^	0.085	0.001
Ω3 (%)	0.74 ^b^	31.91 ^a^	0.060	0.001
Ω6 (%)	4.09 ^b^	21.46 ^a^	0.025	0.001
Ω6/Ω3	5.54 ^a^	0.67 ^b^	0.041	0.001

AE—allicin extract; GL—garlic leaves. Means within a row with no common superscript show significant difference (*p* < 0.05). Different letters (a, b) in the same row show significant differences (*p* < 0.05); SEM—standard error of the mean; *n* = 3.

**Table 3:** Productive parameters registered through experimental period.

ITEM	FO	OO	OOA	OOG	SEM	*p* Value
BWi 14 days (g)	377.4	372.6	375.3	379.7	5.902	0.852
BWf 42 days (g)	3191	3081	3142	3102	45.76	0.367
ADFI (g/day)	164.1	161.4	147.7	157.0	12.36	0.796
ADWG (g/day)	99.95	96.45	98.20	96.68	1.462	0.324
FCR (g/g)	1.642 ^a^	1.673 ^a^	1.504 ^b^	1.624 ^a^	0.024	0.001
Viability (%)	100	100	100	100	-	-
EPEF	462.7	438.5	594.1	454.8	-	-
EBI	608.7	576.5	652.9	595.3	-	-

FO—group fed with fresh oil diet; OO—group fed with oxidized oil; OOA—group fed with oxidized oil and allicin extract; OOG—group fed with oxidized oil and garlic leaves; BWi—initial body weight; BWf—final body weight; ADFI—average daily feed intake; ADWG—average daily weight gain; FCR—feed conversion ratio, representing the ratio between feed intake and weight gain; EPEF—European Production Efficiency Factor; the EBI—European Broiler Index. Different letters (a, b) in the same row show significant differences (*p* < 0.05). SEM—standard error of the mean.

**Table 4:** Proximate and mineral composition of breast and thigh meat samples.

ITEM	FO	OO	OOA	OOG	SEM	*p* Value
Breast samples						
Dry matter (%)	26.73	26.84	27.08	26.86	0.294	0.860
Crude protein (%)	83.11	81.80	82.42	81.92	1.024	0.797
Crude fat (%)	4.868	5.771	6.012	5.655	0.634	0.616
Crude ash (%)	4.040	3.978	3.983	4.007	0.042	0.717
Fe (mg/kg)	17.66 ^b^	21.51 ^a^	20.28 ^ab^	23.35 ^a^	0.859	0.001
Zn (mg/kg)	21.75	22.80	21.39	20.77	0.795	0.358
Thigh samples						
Dry matter (%)	23.78 ^b^	24.06 ^b^	25.63 ^a^	24.67 ^ab^	0.388	0.015
Crude protein (%)	75.57	75.64	69.71	68.42	2.888	0.191
Crude fat (%)	12.56 ^b^	13.00 ^b^	18.58 ^a^	14.87 ^ab^	1.259	0.012
Crude ash (%)	4.133 ^ab^	4.143 ^ab^	3.932 ^b^	4.308 ^a^	0.068	0.009
Fe (mg/kg)	10.11 ^b^	14.81 ^a^	10.39 ^b^	14.47 ^a^	1.280	0.023
Zn (mg/kg)	54.47	51.83	49.61	53.96	1.933	0.295

FO—group fed with fresh oil diet; OO—group fed with oxidized oil; OOA—group fed with oxidized oil and allicin extract; OOG—group fed with oxidized oil and garlic leaves. Means within a row with no common superscript show significant difference (*p* < 0.05). Different letters (a, b) in the same row show significant differences (*p* < 0.05). SEM—standard error of the mean.

**Table 5:** Fatty acid composition of breast meat samples.

ITEM	FO	OO	OOA	OOG	SEM	*p* Value
Breast meat samples						
Butyric acid (%)	0.000	0.004	0.025	0.017	0.006	0.047
Caproic acid (%)	0.062 ^ab^	0.097 ^a^	0.049 ^ab^	0.041 ^b^	0.013	0.030
Caprylic acid (%)	0.074 ^ab^	0.094 ^a^	0.047 ^b^	0.059 ^ab^	0.011	0.047
Capric acid (%)	0.066 ^ab^	0.098 ^a^	0.039 ^b^	0.050 ^b^	0.011	0.004
Lauric acid (%)	0.058	0.044	0.041	0.022	0.013	0.312
Myristic acid (%)	0.442	0.567	0.495	0.508	0.034	0.102
Myristioleic acid (%)	0.059	0.091	0.064	0.088	0.014	0.273
Pentadecanoic acid (%)	0.046	0.062	0.033	0.047	0.011	0.340
Pentadecenoic acid (%)	0.096 ^a^	0.055 ^b^	0.053 ^b^	0.045 ^b^	0.009	0.003
Palmitic acid (%)	17.14 ^c^	21.28 ^a^	18.64 ^bc^	19.50 ^ab^	0.516	0.001
Palmitoleic acid (%)	2.670	3.918	2.399	3.174	0.468	0.142
Heptadecanoic acid (%)	0.138 ^b^	0.136 ^b^	0.203 ^a^	0.198 ^a^	0.012	0.001
Heptadecenoic acid (%)	0.060	0.057	0.056	0.051	0.011	0.940
Stearic acid (%)	6.985	7.933	8.164	7.465	0.315	0.069
Oleic acid (%)	36.51 ^a^	30.22 ^b^	28.41 ^b^	30.51 ^b^	1.175	0.001
Linoleic cis acid (%)	25.78 ^b^	27.35 ^ab^	31.61 ^a^	30.41 ^a^	1.099	0.004
Linolenic acid (%)	0.006	0.006	0.017	0.016	0.005	0.277
Arachic acid (%)	0.157	0.173	0.204	0.238	0.029	0.248
Linolenic α acid (%)	2.077 ^a^	0.410 ^b^	0.457 ^b^	0.491 ^b^	0.164	0.001
CLA (%)	0.186	0.171	0.086	0.128	0.034	0.175
Octadecatetraenoic acid (%)	0.392 ^a^	0.198 ^b^	0.300 ^ab^	0.283 ^ab^	0.039	0.019
Eicosadienoic acid (%)	0.318	0.265	0.164	0.188	0.049	0.135
Eicosatrienoic acid (%)	0.697	0.744	0.792	0.759	0.060	0.732
Erucic acid (%)	0.082	0.096	0.064	0.068	0.021	0.706
Eicosatrienoic acid (%)	0.576	0.552	0.542	0.498	0.028	0.273
Arachidonic acid (%)	3.280 ^ab^	3.198 ^ab^	3.892 ^a^	2.748 ^b^	0.226	0.017
Docosadienoic acid (%)	0.001 ^b^	0.038 ^ab^	0.086 ^a^	0.090 ^a^	0.015	0.001
Docosatrienoic acid (%)	0.001 ^b^	0.040 ^ab^	0.074 ^a^	0.077 ^a^	0.017	0.014
Eicosapentaenoic acid (%)	0.031 ^b^	0.126 ^a^	0.103 ^ab^	0.097 ^ab^	0.020	0.016
Lignoceric acid (%)	0.032 ^b^	0.248 ^a^	0.167 ^ab^	0.154 ^ab^	0.039	0.008
Nervonic acid (%)	1.042 ^ab^	0.995 ^b^	1.397 ^a^	0.975 ^b^	0.095	0.016
Docosatetraenoic acid (%)	0.203 ^b^	0.133 ^b^	0.389 ^a^	0.220 ^b^	0.037	0.001
Docosapentaenoic acid (%)	0.127	0.112	0.119	0.112	0.017	0.899
Docosahexaenoic acid (%)	0.064	0.058	0.077	0.075	0.013	0.675
Other fatty acids	0.548	0.427	0.741	0.592	-	-
SFAs (%)	25.19 ^b^	30.73 ^a^	28.11 ^a^	28.30 ^a^	0.691	0.001
MUFAs (%)	40.51 ^a^	35.43 ^ab^	32.44 ^b^	34.91 ^b^	1.313	0.003
PUFAs (%)	33.74 ^b^	33.40 ^b^	38.71 ^a^	36.19 ^ab^	1.213	0.020
Ω3 (%)	3.268 ^a^	1.456 ^b^	1.600 ^b^	1.556 ^b^	0.194	0.001
Ω6 (%)	30.28 ^b^	32.11 ^ab^	37.02 ^a^	34.51 ^ab^	1.307	0.009

FO—group fed with fresh oil diet; OO—group fed with oxidized oil; OOA—group fed with oxidized oil and allicin extract; OOG—group fed with oxidized oil and garlic leaves; SFAs—saturated fatty acids; MUFAs—monounsaturated fatty acids; PUFAs—polyunsaturated fatty acids. Different letters (a, b, c) in the same row show significant differences (*p* < 0.05). SEM—standard error of the mean.

**Table 6:** Fatty acid composition of thigh meat samples.

ITEM	FO	OO	OOA	OOG	SEM	*p* Value
Thigh meat samples						
Butyric acid (%)	0.149 ^b^	0.185 ^b^	0.407 ^a^	0.235 ^ab^	0.053	0.013
Caproic acid (%)	0.229	0.277	0.341	0.227	0.046	0.287
Caprylic acid (%)	0.445 ^a^	0.377 ^ab^	0.307 ^b^	0.115 ^c^	0.021	0.001
Capric acid (%)	0.309 ^a^	0.252 ^a^	0.121 ^b^	0.064 ^b^	0.015	0.001
Lauric acid (%)	0.024	0.022	0.065	0.060	0.018	0.210
Myristic acid (%)	0.951 ^a^	0.899 ^a^	0.668 ^b^	0.603 ^b^	0.034	0.001
Myristoleic acid (%)	0.094	0.096	0.078	0.101	0.010	0.453
Pentadecanoic acid (%)	0.156	0.152	0.226	0.166	0.030	0.290
Pentadecenoic acid (%)	0.567 ^a^	0.249 ^b^	0.259 ^b^	0.342 ^ab^	0.061	0.005
Palmitic acid (%)	20.61	21.53	20.47	21.71	0.475	0.186
Palmitoleic acid (%)	3.590	3.867	3.355	3.945	0.369	0.664
Heptadecanoic acid (%)	0.184	0.185	0.203	0.191	0.014	0.752
Heptadecenoic acid (%)	0.141	0.094	0.102	0.124	0.019	0.329
Stearic acid (%)	7.918	7.812	8.083	7.419	0.281	0.408
Oleic acid (%)	31.10	30.92	30.32	32.31	0.770	0.342
Linoleic cis acid (%)	25.21	25.15	27.39	24.73	1.263	0.459
Linolenic acid (%)	0.027	0.024	0.024	0.045	0.013	0.609
Arachic acid (%)	0.195	0.185	0.191	0.168	0.026	0.889
Linolenic α acid (%)	0.383	0.329	0.337	0.316	0.028	0.379
CLA (%)	0.077 ^a^	0.022 ^b^	0.069 ^a^	0.014 ^b^	0.024	0.020
Octadecatetraenoic acid (%)	0.432	0.576	0.454	0.541	0.074	0.471
Eicosadienoic acid (%)	0.441	0.619	0.431	0.546	0.052	0.055
Eicosatrienoic acid (%)	0.530 ^a^	0.456 ^ab^	0.391 ^b^	0.369 ^b^	0.030	0.005
Erucic acid (%)	0.097	0.057	0.033	0.044	0.018	0.112
Eicosatrienoic acid (%)	0.409 ^a^	0.354 ^ab^	0.267 ^b^	0.300 ^ab^	0.031	0.020
Arachidonic acid (%)	2.915	2.354	2.370	1.886	0.270	0.096
Docosadienoic acid (%)	0.240	0.305	0.322	0.396	0.043	0.122
Docosatrienoic acid (%)	0.230 ^b^	0.279 ^ab^	0.373 ^ab^	0.379 ^a^	0.038	0.027
Eicosapentaenoic acid (%)	0.480	0.488	0.521	0.460	0.049	0.111
Lignoceric acid (%)	0.404	0.500	0.566	0.566	0.060	0.214
Nervonic acid (%)	0.764	0.619	0.551	0.503	0.086	0.186
Docosatetraenoic acid (%)	0.232	0.110	0.110	0.173	0.061	0.454
Docosapentaenoic acid (%)	0.045	0.045	0.121	0.082	0.033	0.316
Docosahexaenoic acid (%)	0.027 ^b^	0.003 ^c^	0.028 ^b^	0.102 ^a^	0.012	0.001
Other fatty acids	0.385	0.603	0.438	0.624	-	-
SFAs (%)	31.57	32.38	31.65	31.53	0.625	0.747
MUFAs (%)	36.35	35.90	34.70	37.37	1.024	0.345
PUFAs (%)	31.68	31.11	33.21	30.47	1.425	0.578
Ω3 (%)	1.676	1.794	1.728	1.802	0.091	0.300
Ω6 (%)	29.83	29.30	31.41	28.53	1.436	0.552

FO—group fed with fresh oil diet; OO—group fed with oxidized oil; OOA—group fed with oxidized oil and allicin extract; OOG—group fed with oxidized oil and garlic leaves. Different letters (a, b, c) in the same row show significant differences (*p* < 0.05). SEM—standard error of the mean.

**Table 7:** Lipid quality indices calculated for breast and thigh samples.

Parameter	FO	OO	OOA	OOG	SEM	*p* Value
Nutritional indices						
Breast samples						
PUFA/SFA	1.353 ^a^	1.088 ^b^	1.380 ^a^	1.283 ^ab^	0.060	0.010
Ω6/Ω3	9.954 ^b^	22.04 ^a^	23.18 ^a^	22.48 ^a^	1.199	0.001
(DHA + EPA)/LNA	0.027 ^b^	0.450 ^a^	0.396 ^a^	0.360 ^a^	0.033	0.001
AA/LA	0.127 ^a^	0.117 ^ab^	0.124 ^a^	0.091 ^b^	0.008	0.013
Thigh samples						
PUFA/SFA	1.002	0.965	1.058	0.970	0.061	0.690
Ω6/Ω3	18.84	16.58	18.39	16.08	1.371	0.310
(DHA + EPA)/LNA	0.827 ^b^	1.562 ^ab^	1.670 ^ab^	2.002 ^a^	0.251	0.024
AA/LA	0.116 ^a^	0.093 ^ab^	0.085 ^ab^	0.076 ^b^	0.008	0.018
Health indices						
Breast samples						
AI	0.257 ^c^	0.342 ^a^	0.291 ^bc^	0.304 ^ab^	0.011	0.001
TI	0.549 ^c^	0.780 ^a^	0.690 ^b^	0.697 ^b^	0.025	0.001
OFAs	17.58 ^c^	21.85 ^a^	19.13 ^bc^	20.01 ^ab^	0.538	0.001
DFAs	81.24 ^a^	76.77 ^c^	79.32 ^ab^	78.57 ^bc^	0.544	0.001
Thigh samples						
AI	0.331 ^ab^	0.365 ^a^	0.327 ^b^	0.341 ^ab^	0.009	0.034
TI	0.655 ^b^	0.793 ^a^	0.738 ^a^	0.754 ^a^	0.018	0.001
OFAs	21.56	22.43	21.14	22.32	0.496	0.234
DFAs	81.24 ^a^	76.77 ^c^	79.32 ^ab^	78.57 ^bc^	0.544	0.001
Oxidative indices						
Breast samples						
PI	51.57 ^ab^	48.44 ^b^	57.31 ^a^	50.67 ^ab^	1.793	0.015
IV	84.02 ^a^	78.16 ^b^	82.66 ^ab^	83.21 ^a^	1.171	0.009
COX	3.471 ^ab^	3.209 ^b^	3.643 ^a^	3.547 ^ab^	0.103	0.043
OS	5700 ^a^	2162 ^b^	2480 ^b^	2498 ^b^	348.3	0.001
Thigh samples						
PI	47.31	46.38	48.51	44.98	2.031	0.661
IV	74.83	74.69	77.58	75.21	1.506	0.504
COX	2.997	2.976	3.202	2.949	0.127	0.489
OS	2060	1930	2045	1928	100.9	0.685

FO—group fed with fresh oil diet; OO—group fed with oxidized oil; OOA—group fed with oxidized oil and allicin extract; OOG—group fed with oxidized oil and garlic leaves; AI—atherogenicity index; TI—thrombogenicity index; OFAs—hypercholesterolemic fatty acids; DFAs—hypocholesterolemic fatty acids; PI—peroxidisability index; IV—iodine value; COX—calculated oxidizability; OS—oxidative susceptibility. Different letters (a, b, c) in the same row show significant differences (*p* < 0.05). SEM—standard error of the mean.

## Data Availability

The original contributions presented in the study are included in the article, and further inquiries can be directed to the corresponding author.
